# V-Scar Lift: An Innovative Technique for Treating the Lower Third of the Face and the Upper Neck

**DOI:** 10.1007/s00266-025-05564-7

**Published:** 2026-01-21

**Authors:** Giorgos Andreanidis, Ioanna Andreanidi, Giorgos Eustathiou, Pantelis Diamantopoulos

**Affiliations:** 1 Maria Hilf Hospital: Krakenhaus Maria Hilf, Hospitalstrabe 3-7, 59581 Warstein, Germany; 2 Medical University of Sofia, Boulevard “Akademik Ivan Evstratiev Geshov” 15, 1431 Sofia, Bulgaria; 3 Erythros Stavros Hospital, Athanasaki 11, 11526 Athens, Greece; 4 Ethniko and Kapodistriako University of Athens, Mikras Asias 75, Goudi, 11527 Athens, Greece

**Keywords:** Face lift, Neck lift, Jowls correction, Mini face lift, V-Scar Lift

## Abstract

**Background:**

A novel minimally invasive surgical technique is proposed to treat the contour of the lower face, jowls and upper neck. The procedure is mainly used in patients who wish to undergo less extended surgery, with minimal scarring and less downtime.

**Methods:**

In the described technique, a V-shaped incision is made between the middle of the tragus and the insertion point of the posterior auricular muscle at the back of the auricle. The incision goes around the earlobe. The skin in a radius of 4 cm around the earlobe is undermined with Metzenbaum or Solz scissors. The platysma below the mandibular angle is also undermined 3-4 cm. Then, we plicate the superficial musculoaponeurotic system (SMAS) with permanent sutures. The excess skin is excised, and the final skin sutures are made. No drains are placed.

**Results:**

The technique was successfully applied to 132 consecutive patients over a period of 2.5 years. There was no downtime, no pain and no facial edema after the operation in the majority of the patients. No major complications occurred during the follow-up period. The satisfaction of most patients was extremely high. A follow-up of at least 2 years after the operation indicates the long-lasting results of the operation.

**Conclusion:**

The authors present a simple, effective and safe approach to correct soft tissue ptosis and skin laxity in the lower third of the face and upper neck. General anesthesia is not required, there is no downtime, patient discomfort is minimal, there are very few complications and the results are satisfactory.

**Level of Evidence IV:**

This journal requires that authors assign a level of evidence to each article. For a full description of these Evidence-Based Medicine ratings, please refer to the Table of Contents or the online Instructions to Authors www.springer.com/00266.

## Introduction

Facial aging is a complex process that involves changes in the skin, soft tissues and bone structure. Reduced production of collagen and elastin, decreased levels of hyaluronic acid, fat dedistribution and facial bones shrinking are processes that lead to wrinkles, fine lines, sagging skin and volume loss [1]. All these aspects make the face look older and aged, which usually alters self-perception, with emotional impacts. A more youthful face is combined with positive feelings, self-confidence and a better attitude in interpersonal relationships.

Face lifts are surgical procedures designed to reduce the visible signs of aging in the face and neck. They are extended surgical operations who are conducted under general anesthesia. There are many different surgical techniques such as subcutaneous face lift with SMAS plication, Minimal Access Cranial Suspension (MACS) Short-scar Face lift, Lateral SMASectomy, High SMAS Face lift and Deep Plane variant [2, 3]. It has been constantly emphasized how important is to plicate or undermine the SMAS in order to have the appropriate traction and be able to pull the tissues to the right vector. The superficial musculoaponeurotic system (SMAS) is a well-defined portion of the superficial facial fascia and is essential to work it through the operation in order to obtain the desired result. Lately, the deep plane technique gains more territory in plastic surgery practice [4]. It is based on raising a composite flap of skin and muscle and releasing the zygomatic, masseteric and mandibular ligaments [5].

All these surgical techniques have some important drawbacks such as long operative hours and exposing the patients to the risks of general anesthesia. Additionally, there is pain, discomfort and facial edema after the operation which takes days to resolve. The patient has to wear an elastic band postoperatively, and social activities are restrained. There is also the risk of major surgical complications such as hematoma, great auricular nerve injury, facial nerve injury, seromas, infections and skin sloughs [6]. For all these reasons, there are many patients who do not wish to proceed to such a complex operation and seek minimally invasive techniques with less pain, downtime and scars [7]. This is something that a plastic surgeon has to respect and seek for solutions that do not involve general anesthesia and hospitalization.

The “Mini Face Lift” is an option that addresses this issue and gives dramatic improvement, while minimizing the post-operative complications, in people who desire a less extended, less invasive face lift [8]. We have used the “V-Scar Lift” for 132 patients over the past 2,5 years, correcting the lower third of the face and the upper neck, which is the premium cosmetic concern for women and men over 45 years old. Our approach is a combination of SMAS plication along with subplatymal undermining for longer lasting results.

## Materials and Methods

### Surgical Technique

**Fig. 1:**
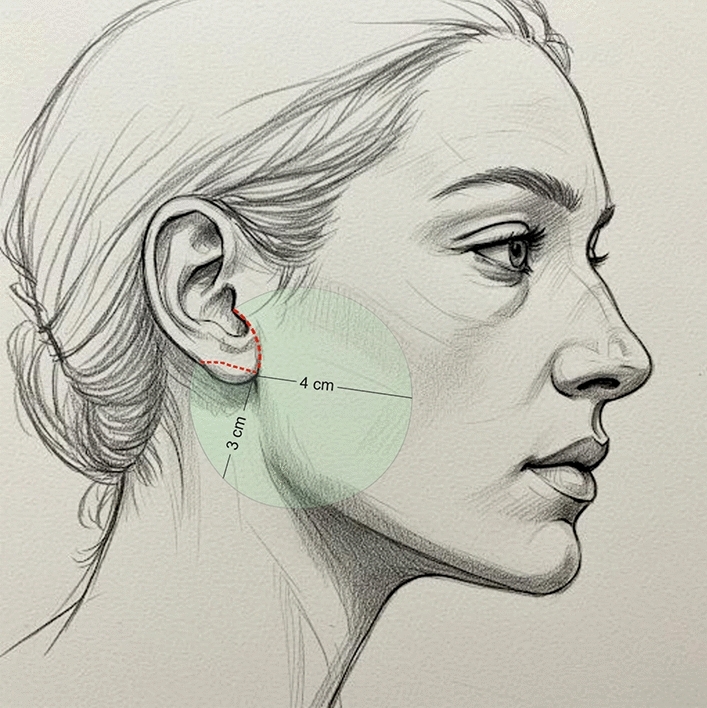
Preoperative markings—extent of the incision (red dot line) and area of undermining (green circle) are shown

**Fig. 2:**
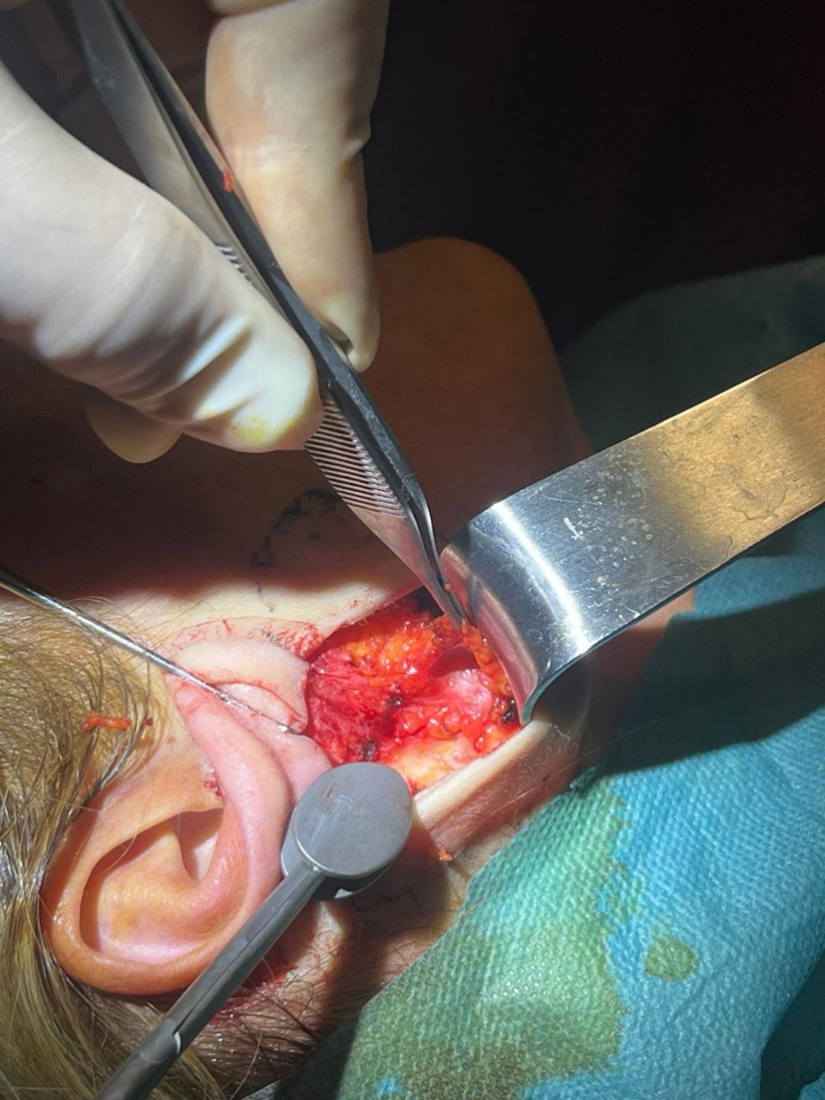
The platysma is undermined using Trepsat scissors

**Fig. 3:**
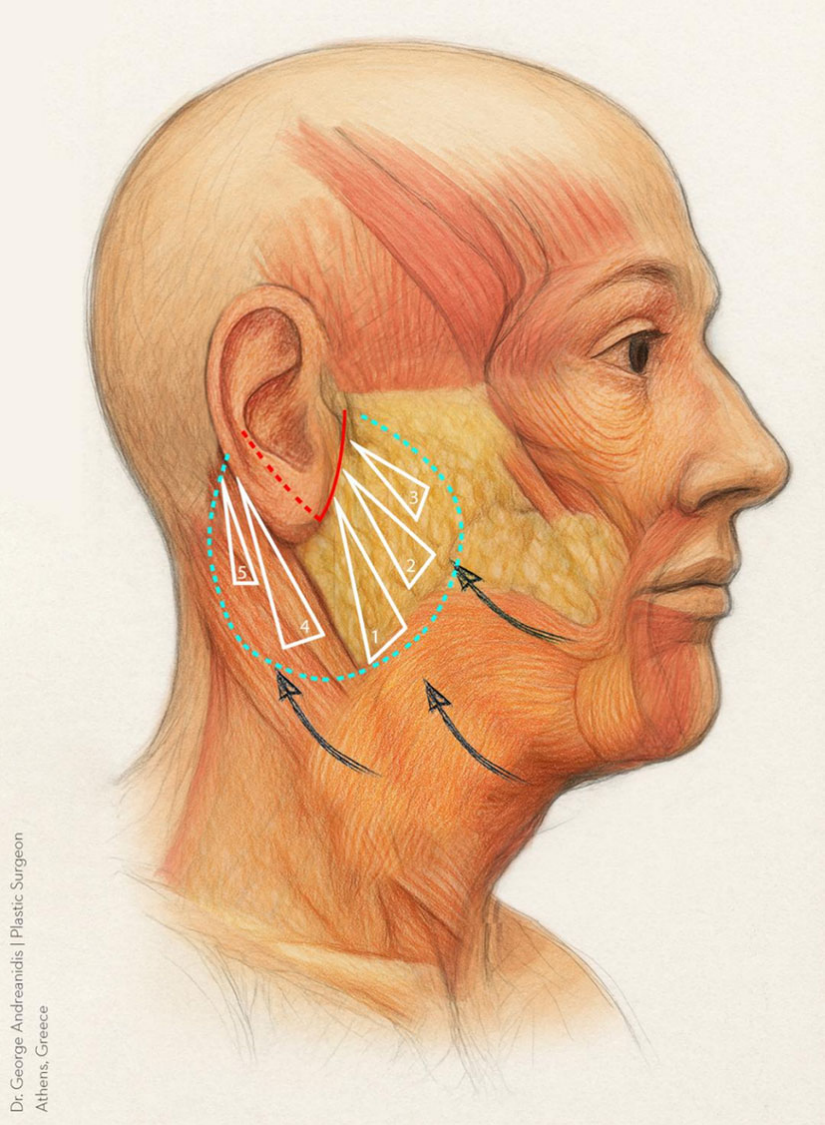
We use 5 distinctive plication points

**Fig. 4:**
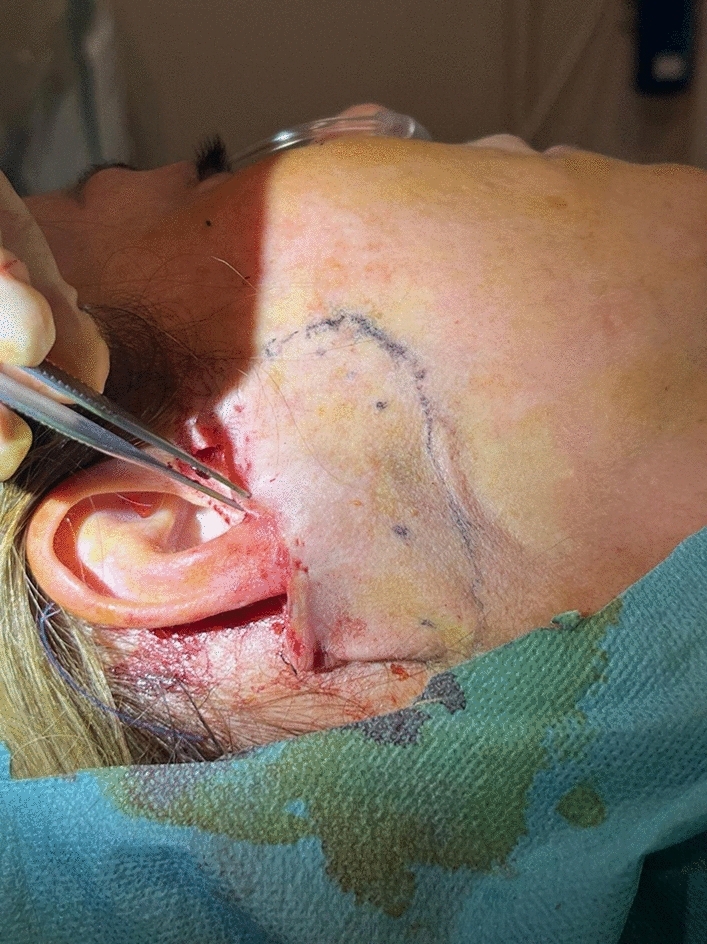
The extra skin is removed and the skin edges are stitched

All the procedures are performed using sedation and local anesthesia. After the patient is draped and sedated, a tumescent solution with 2% lidocaine, 1:1000 epinephrine in normal saline is infiltrated bilaterally (7 cc on each side of the face). Antibacterial prophylaxis is done with injection of 1 gr cephalosporin during the surgery. We also give hydrocortisone intravenously in order to reduce post-operative edema. A V-shaped incision is made. It is extended from the point half of the height of the tragus until the equivalent point at the back of the ear, usually at the level of the insertion of the posterior auricular muscle (Fig. 1). The skin around the earlobe is undermined in a 4-cm radius in front of the ear and 3-3,5 cm at the back using a 15 blade scalpel for the first 1 cm and then Metzenbaum or Solz scissors for the rest of the dissection (Fig. 1). Appropriate hemostasis is conducted with bipolar cautery. The platysma under the mandibular corner is then undermined for 3–4 cm with Trepsat scissors, technique that is also used in deep plane face lift, allowing for a more extensive lifting and tightening of the platysma (Fig. 2). The first non-absorbable suture 2-0 nylon is placed between the platysma, under the mandible corner, and Lore’s fascia at the front of the ear (Fig. 3). The second suture is performed between the SMAS and the Lore’s fascia pre-auricularly. The third suture between the SMAS and the Lore’s fascia close to the tragus. A fourth and fifth suture are placed between the platysma and the mastoid bone. In all the stitches, we take 2 bites to the tissue and the knots are buried under the SMAS. Subsequently, a double, in 2 opposite directions, over and over suture with 3-0 nylon is placed over the simple non-absorbable sutures in order to secure the vector. Then, the extra skin is removed and the skin edges are stitched with non-absorbable sutures (Fig. 4). Drains are not used. There is no need for elastic facial band.

Postoperatively pain killers and antibiotics are prescribed to the patient. They usually do not complain of any pain after the operation. Liquid soft diet is recommended during the surgery day and normal diet from the next day. The follow-up involves, checking the patients on the 1 st and 2nd postoperative day, on the 9th day for skin stitch removal and then on the 3rd, 12th, 18th and 24th month.

### Patients

Over a 2.5 year period, 132 consecutive patients underwent surgery with this technique. All patients had prominent jowls, sagging of the lower face and upper neck skin laxity. Their ages ranged from 37 to 75 years old (mean age 49 years). We excluded heavy smokers (over 20 cigarettes daily) because of the increased risk of wound healing problems. Patients who had previously done biostimulators, threads and energy based devices more than once were also excluded. These people develop subcutaneous fibrosis, which makes skin undermining challenging, and post-operative edema that takes days to resolve. The follow-up period ranged from 1 month to 2.5 years. More specifically, we had a follow-up of 2.5 years for 17 patients, 2 years for 60 patients, 14–16 months for 20 patients and 1–4 months for 16 patients. The remaining 19 patients were not consistent with the follow-up appointments. Almost all of the patients were women. There was only one case of a male patient.

## Results

**Fig. 5:**
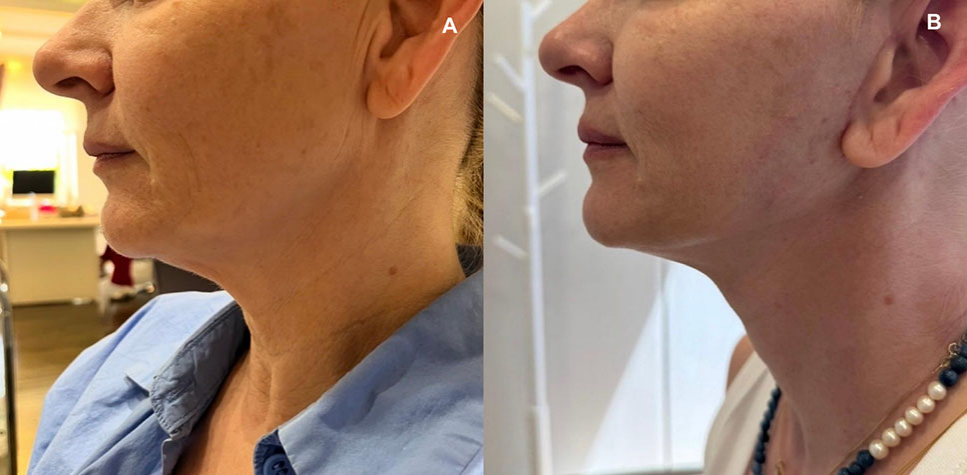
View of a middle aged woman. **A** before and **B** 8 months after the operation

**Fig. 6:**
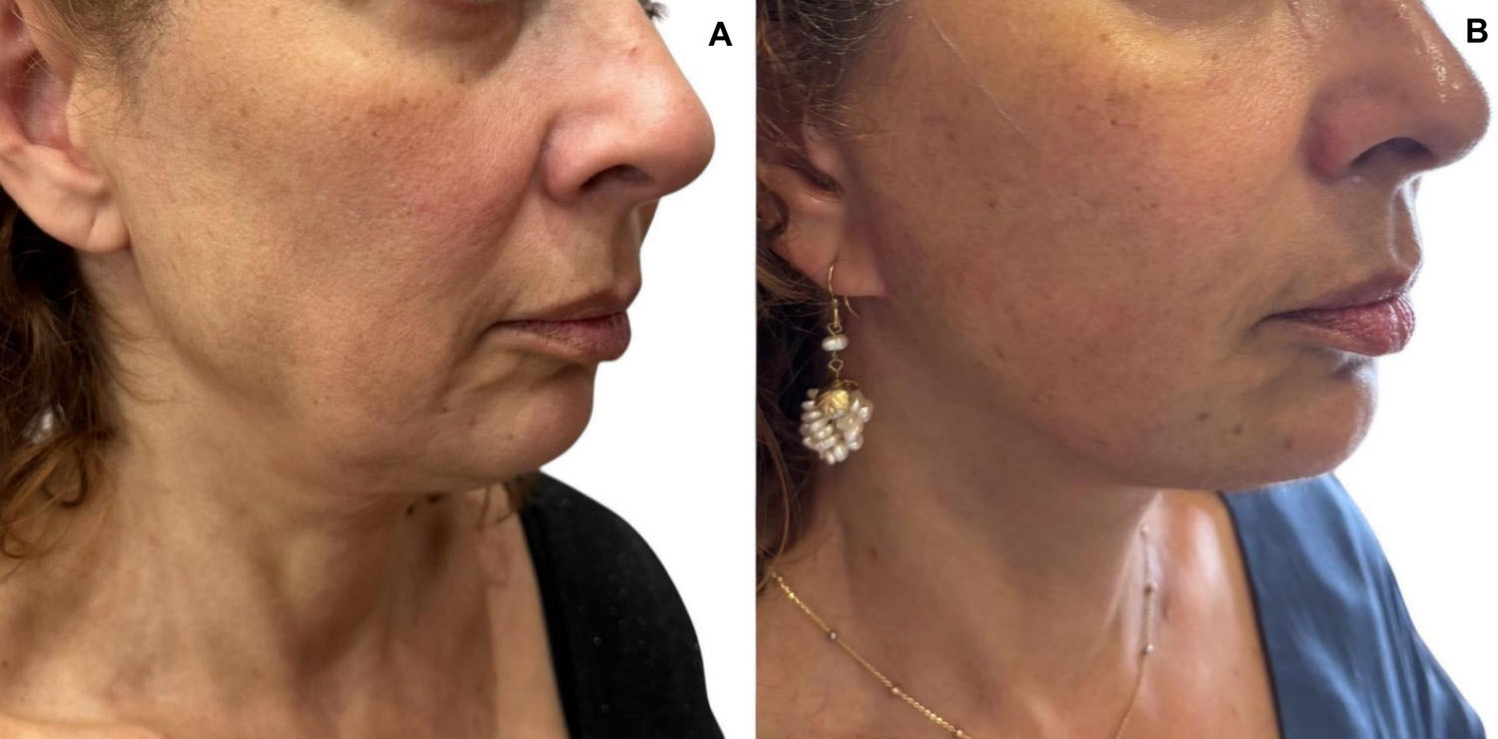
View of another patient. **A** before and **B** 17 months after the operation

**Fig. 7:**
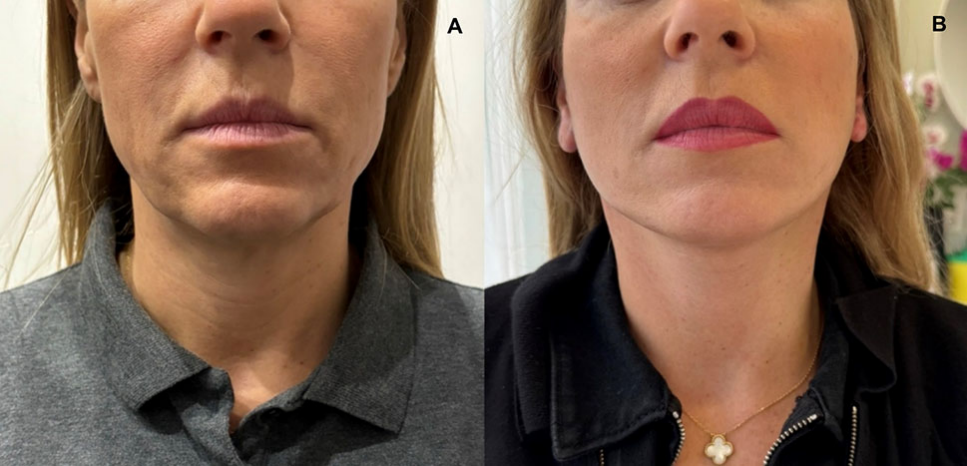
Views of a 48-year old woman. **A** before and **B** 6 months after the operation

**Fig. 8:**
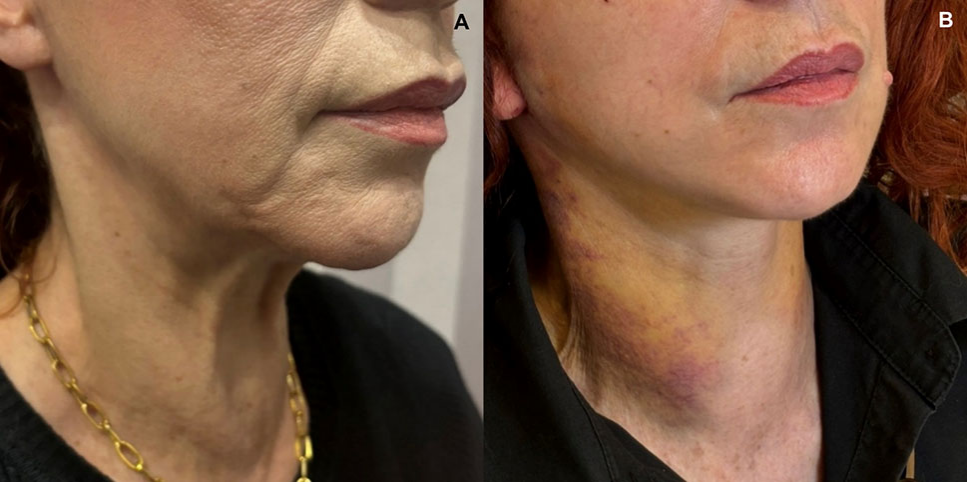
Views of a 59-year old woman. **A** before and **B** after stitches removal

**Fig. 9:**
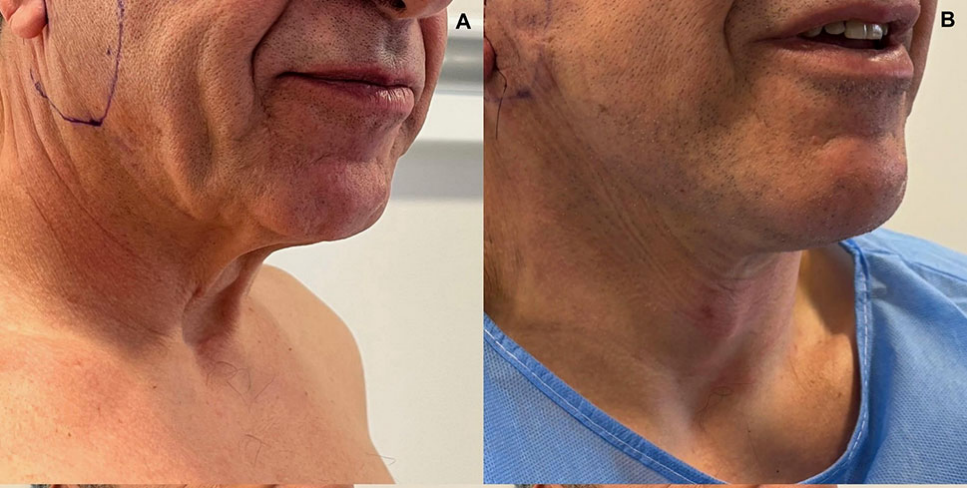
Views of a 60-year old male patient. **A** before and **B** 18 h after the operation

**Fig. 10:**
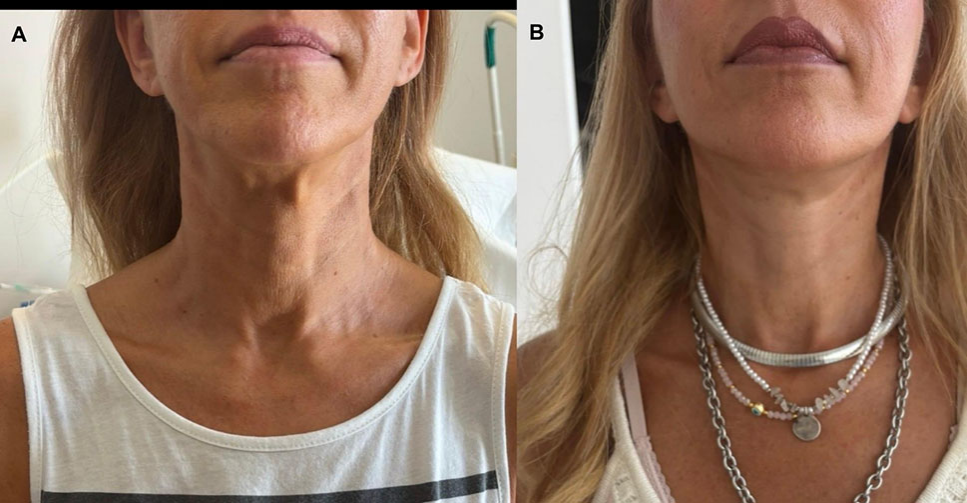
Frontal view of a patient’s neck. **A** before and **B** 5,5 months after the operation, there is no recurrence of platysmal bands

Substantial aesthetic improvement of the lower third of the face and the upper neck was obtained in all cases. All the patients reported improvement to complete elimination of the prominent jowls and the upper neck laxity (Figs. 5, 6, 7, 8). There was also significant improvement of the platysma bands (Figs. 7, 8, 9, 10). There was zero downtime and no pain after the operation. There was no facial edema, only perilobular edema 2 cm radially that was camouflaged with the hair and lasted for 3–6 days. Post-operative scar was rarely visible.

Very few complications were reported mainly in smokers over 40 years old. There were 2 cases of wound dehiscence 5 x 12 mm. Other complications involved 2 cases of post-auricular hypertrophic scar, that was surgical corrected 3 months after the operation. There were also 4 cases of perilobular edema in all the undermined area that lasted 8–10 days and was easily camouflaged with hair. The particular patients were previously submitted to many injectable and thread therapies as well as energy based devices like Hi-Fu. No major complications, like hematomas, nerve injury and irregularities occurred. Relapses were not observed during the follow-up period.

## Discussion

Face lift is a surgical procedure that aims to lift and tighten the soft tissues of the face and rejuvenate someone’s appearance [9]. It reduces the deep wrinkles, corrects jowls, improves nasolabial folds and naturally volumizes the middle third of the face [7, 9]. There are several techniques being proposed by various authors such as High Smas Lift, Lateral SMASectomy, Macs Lift and Subperiosteal Face lift [2, 10]. Lately, the deep plane face lift is a technique that promises to give better results, especially in older patients [9]. It relies on lifting a composite skin-smas flap and extended release of facial ligaments, such as the zygomatic, the masseteric and the mandibular ligaments. Although it is considered a safer, regarding skin necrosis, technique because of the better vascularity of the composite flap, has the drawback of putting the facial nerve branches at risk even with experienced surgeons [11].

All these techniques are extended, time-consuming operations, require general anesthesia and have a long downtime. There are many patients that seek for a less invasive technique with shorter downtime [12]. Others have comorbidities, and they wish to avoid the risks of general anesthesia. All of these reasons along with the cost and the risk of an overcorrected look make the mini face lift an alternative for many patients [13].

In order to address these issues, we propose a V-shaped scar mini face lift that mainly treats the lower third of the face and the upper neck. A V-incision around the earlobe is used, and the skin is undermined in a 4-cm radius (Fig. 1). The platysma is undermined also with Trepsat scissors for 3–4 cm (Fig. 2). The SMAS and the platysma are pulled with non-absorbable sutures and stitched to stable points like the Lore’s fascia and the periosteum of the mastoid (Fig. 3). The extra skin is incised, and the skin edges are stitched with continuous half buried seam post auricularly and continuous intradermal in front of the ear (Fig. 4). A 4-0 nylon suture is used.

All the operations are being held in an office’s setting with sedation and local anesthesia. They usually last about 1 or 2 h with a mean time of 1 h and 15 min, which is increased if additional cosmetic surgical procedures are used. There are no drains, patients do not need hospitalization, and they go home after the operation. None of the patients complained about post -operative pain, and there was no need for painkillers.

Technically, we found difficulties only with patients that had previously done anti-aging therapies like threads, mesotherapies, biostimulators like poly-L-lactic acid (PLLA), hydroxyapatite and other injectables and energy based devices as well. The undermining of the skin was extremely difficult in these cases because of the hard scar tissue due to previous collagenesis. Perilobular edema was observed in 80% of these patients that took 8–10 days to resolve. This complication didn’t obstruct their daily activities because the swelling was easily hidden behind the hair.

Like more extended face lifts, the patients are advised to smoking cessation 1 month before and 1 month after the operation, but smokers do not usually comply [13]. However, smoking-related complications like infections and necrosis weren’t observed.

Regarding preoperative checkup, all patients are submitted to general blood test and INR. Moreover, they all have their blood pressure checked and controlled in order to avoid post-surgical complications like hematoma [14]. During the operation, all patients are connected with monitor, and the vital signs along with the heart rate are regularly checked by the anesthesiologist.

**Fig. 11:**
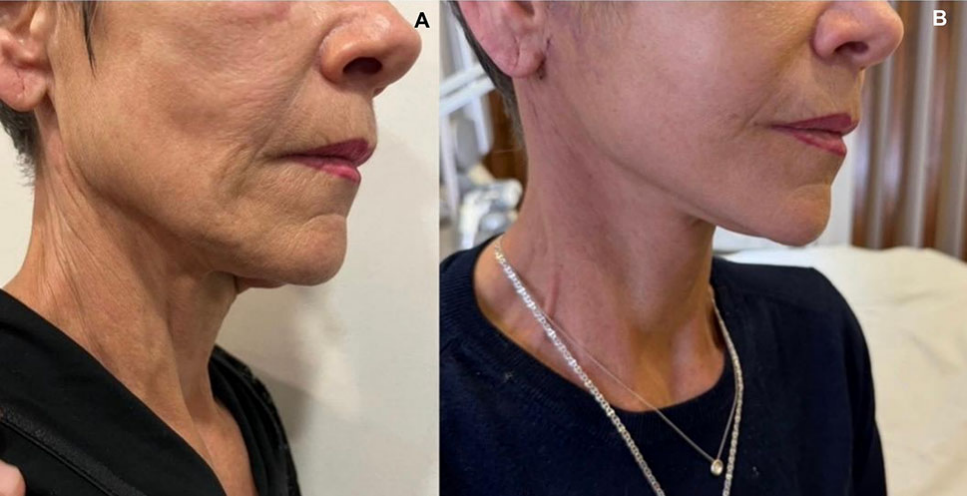
View of a patient. **A** before and **B** 10 months after V-Scar Lift and fat grafting to the middle face

If indicated, fat grafting is usually performed simultaneously, as an adjunctive procedure, to improve the outcomes of V-Scar Lift and to enhance the contour of the zygomatic bone [15]. Additional areas that are usually refilled with fat grafting are temples, lower lids and chin (Fig. 11). Other surgical procedures combined with the Mini V-Lift include upper and lower blepharoplasty and Mini Subcutaneous Brow Lift [16]. All these procedures improve the surgical outcomes of the Mini V-Scar Lift. In our series of 132 cases, we combined the 35 with fat grafting and the 32 with brow lift and upper blepharoplasty.

Like every cosmetic surgical procedure follow-up is really important to evaluate the efficiency of the technique and the durability of results. Extended face lift offers long-term results with the jowl, nasolabial and marionette areas remaining well corrected at 5.5 years follow-up but with partial relapse of neck correction [17]. In our series, we had a follow-up of about 2 years. The 75 patients were extremely happy with the result. All of them are pre-operatively informed that the results of V-Scar Lift are not comparable with those of a full face and neck lift lasting about 4–5 years. However, all patients are happy with the less extended scar, less operative time, minimum downtime and post-surgical pain and the cosmetic effect on the lower third of the face and upper neck. Having the experience of the mini face lift and its good results, many of them feel more confident to consider a full face and neck lift as an option for the future. A more extended face–neck lift would address the sagging of a middle, lower face and the whole neck, eliminate the platysma bands and efficiently lift the face in a more vertical vector.

Despite demonstrating promising initial outcomes, such as enhanced jawline definition, reduced jowling and alleviated platysma bands (Figs. 5, 6, 7, 8, 9, 10, 11) with our novel V-Scar Lift technique, several limitations warrant acknowledgment. First, the relatively small sample of 132 patients restricts the generalizability of our findings. A larger, multicenter study would be necessary to validate these results. Secondly, the follow-up period of 2.5 years is relatively short for assessing the long-term durability of aesthetic outcomes in facial rejuvenation surgery. Future investigations with more extended follow-up will be critical to determine the longevity of improvements achieved with this technique. Additionally, while subjective patient satisfaction was high, the absence of objective quantitative measurements limited our ability to precisely quantify changes in facial volume, skin laxity or specific anatomic vectors. The implementation of patient-reported outcome measures (PROMS) or standardized photographic grading scales in subsequent studies would provide a more robust assessment of surgical efficacy.

## Conclusion

The minimally invasive V-Scar Lift is a safe alternative that respects the facial harmony and provides less morbidity and complications, achieves durable results and offers a great degree of satisfaction to patients. The surgical procedure is relatively simple to perform, with less scars, pain and downtime and achieves optimal results for the tightening of the lower third of the face and the upper neck.
